# Benchmarking the quality of breast cancer care in a nationwide voluntary system: the first five-year results (2003–2007) from Germany as a proof of concept

**DOI:** 10.1186/1471-2407-8-358

**Published:** 2008-12-02

**Authors:** Sara Y Brucker, Claudia Schumacher, Christoph Sohn, Mahdi Rezai, Michael Bamberg, Diethelm Wallwiener, the Steering Committee

**Affiliations:** 1 Department of Obstetrics and Gynaecology, University of Tübingen, Tübingen, Germany; 2 St. Elisabeth-Krankenhaus Köln-Hohenlind, Cologne, Germany; 3 Department of Obstetrics and Gynaecology, University of Heidelberg, Heidelberg, Germany; 4 Brustzentrum Düsseldorf im Luisenkrankenhaus, Düsseldorf, Germany; 5 Department of Radiooncology, University of Tübingen, Tübingen, Germany; 6 For full list, see Acknowledgements

## Abstract

**Background:**

The main study objectives were: to establish a nationwide voluntary collaborative network of breast centres with independent data analysis; to define suitable quality indicators (QIs) for benchmarking the quality of breast cancer (BC) care; to demonstrate existing differences in BC care quality; and to show that BC care quality improved with benchmarking from 2003 to 2007.

**Methods:**

BC centres participated voluntarily in a scientific benchmarking procedure. A generic XML-based data set was developed and used for data collection. Nine guideline-based quality targets serving as rate-based QIs were initially defined, reviewed annually and modified or expanded accordingly. QI changes over time were analysed descriptively.

**Results:**

During 2003–2007, respective increases in participating breast centres and postoperatively confirmed BCs were from 59 to 220 and from 5,994 to 31,656 (> 60% of new BCs/year in Germany). Starting from 9 process QIs, 12 QIs were developed by 2007 as surrogates for long-term outcome. Results for most QIs increased. From 2003 to 2007, the most notable increases seen were for preoperative histological confirmation of diagnosis (58% (in 2003) to 88% (in 2007)), appropriate endocrine therapy in hormone receptor-positive patients (27 to 93%), appropriate radiotherapy after breast-conserving therapy (20 to 79%) and appropriate radiotherapy after mastectomy (8 to 65%).

**Conclusion:**

Nationwide external benchmarking of BC care is feasible and successful. The benchmarking system described allows both comparisons among participating institutions as well as the tracking of changes in average quality of care over time for the network as a whole. Marked QI increases indicate improved quality of BC care.

## Background

Implementing and maintaining quality assurance procedures and improving cancer care are two key areas of multidisciplinary oncology today. With the high incidence of breast cancer (BC) and the recognised necessity for a multidisciplinary approach to its treatment, the management of this cancer can be considered a prototypical example of an entire process chain ranging from early detection, diagnosis and treatment to follow-up.

Multidisciplinary and intersectoral, i.e. in- and out-patient, BC care requires elements of quality management, particularly at various interfaces along the process chain. In addition, maintaining a quality management system (QMS) with continual quality assurance (QA), which includes both comprehensive documentation of all treatments and external analysis of the QA data, is also one of the prerequisites in Germany for the certification of breast centres [1,2] in accordance with the Requirements of Breast Centres (FAB) [3] set by the German Cancer Society (DKG) and the German Society of Senology (DGS) largely on the basis of two multidisciplinary level-3 guidelines [4,5].

To measure and improve the quality of care provided to BC patients has also in recent years been the main objective of a collaboration established in 2003 between the DGS, DKG and German Society of Obstetrics and Gynaecology (DGGG) on the one hand and the West German Breast Centre (WBC), a subsidiary of the German Oncology Centre (DOC), on the other. The mainstays of this approach are the centralisation of expertise in certified breast centres treating more than 150 primary breast cancer patients per year, and determination of the quality of care at a national level by independent external documentation and analysis of the relevant QA data [1,2,6].

Establishing a nationwide oncology benchmarking system to achieve this goal, however, requires the creation of a suitable infrastructure and appropriate procedures for the collection of uniformly structured, preferably XML-based QA data from the participating institutions and for the analysis of such data. Based on clinical parameters derived from the QA data, quality indicators (QIs) need to be defined as measures of quality of care. To be suitable as QIs, these measures need to reflect the FAB requirements and the degree to which they are achieved by each participating institution. The individual results for each QI can then be used to rank centres (which are coded to protect their reputation) and to determine current mean achievement of the relevant FAB requirements. In addition, mean degrees of achievement of the requirements can be analysed over multi-year periods to determine longer-term changes in QIs and thus in quality of care. In theory, QIs can be defined for all three types of quality – process, structural and outcome quality – but in practice benchmarking of BC care must resort to process QIs as surrogates for outcome quality for at least the first five years. Because of the well-known protracted natural history of breast cancer, QIs representing outcome in terms of morbidity and mortality, e.g. complication and relapse rates and disease-free survival, can be anticipated to require a minimum of five to ten years of data accrual.

The aim of the present study was to establish a comprehensive collaborative network based on voluntary self-declaration of QA data which would ultimately encompass all breast cancer centres in Germany and, eventually, internationally; to develop suitable indicators for benchmarking the quality of care delivered to BC patients, primarily in Germany; and to demonstrate that the quality of cancer care could be assessed, and subsequently improved, by means of standardised collection and analysis of such voluntary QA data by an independent organisation in accordance with the criteria specified by the relevant medical societies.

To this end, the study sought to address a number of key points, the first being to demonstrate in principle the feasibility of voluntary self-declaration of QA data by breast centres in Germany to ascertain the quality of BC care resulting from the introduction of multidisciplinary breast centres, particularly certified centres, and the greater transparency of QA data associated with their establishment. The next points to be verified were whether such QA data could be centrally collected and analysed by an independent external scientific organisation, whether the data were plausible and what indicators of process quality would be suitable as surrogates for outcome quality in order to provide proof of concept for uniform benchmarking of BC care. The last point concerned the extent to which the collection and logical analysis of QA data could be harmonised and conducted homogeneously, and the question whether such a benchmarking system of QIs would indeed reflect the expected continual improvement in the care of breast cancer patients over the first five-year period of the study.

In the following we aim to show on the basis of the data from 2003 to 2007 that it proved possible in Germany to establish a collaboration between a growing supraregional voluntary network of breast centres and an independent provider of external data collection and data analysis services, that self-declaration yielded plausible, uniform QA data, and that the newly implemented benchmarking system proved a successful instrument for quality assurance and indeed reflected improvement in BC care during the study period.

## Methods

### Study design and objectives

The present study was a prospective interventional multicentre feasibility study designed to provide proof of concept that the quality of breast cancer (BC) care in Germany could be measured scientifically and improved by implementing a supraregional benchmarking system based on a set quality indicators (QIs) derived from clinically relevant parameters in accordance with criteria laid down by the relevant scientific medical societies.

The study was prospective in that it was initiated to identify and calculate QIs from collected BC treatment data, and to review and reassess the QIs annually with regard to their suitability as indicators of quality, and of differences in quality, of BC care, with the option of modifying existing QIs or adding new ones, or of abandoning any QIs that lacked discriminatory power so as ultimately to demonstrate in terms of QI increases the postulated improvement in quality of care from benchmarking over a period of several years.

The study was interventional in two respects. Firstly, a collaborative study network of BC centres needed to be established at the outset to enable external collection of structured data sets and to identify suitable QIs for benchmarking quality of treatment and care. Secondly, it was necessary for the purpose of external data collection and independent data analysis to establish collaboration with a provider of such services that was separate from, and independent of, the participating clinical institutions. To this end, a formal collaboration was established with the West German Breast Centre (Westdeutsches Brust-Centrum; WBC), a subsidiary of the German Oncology Centre (Deutsches Onkologie Centrum; DOC), Düsseldorf, Germany.

An additional focus was on developing an XML-based data set structure that would be compatible with any type of breast cancer documentation software and suitable for cancer care quality assurance in order to enable, ensure and enhance uniform data collection and data analysis.

The final objective of the study was to show that supraregional collaborative benchmarking would be associated with continual, marked improvement in the quality of multidisciplinary BC care even during the first five-year period from 2003 to 2007.

### Participating institutions

Hospitals and specialist breast centres in Germany and Switzerland (as of 2006) with a focus on BC care were invited to participate on a voluntary basis in an external, independent, scientific benchmarking system developed by the German Cancer Society (DKG) and the German Society of Senology (DGS) and operated by the WBC/DOC (West German Breast Centre/German Oncology Centre).

### Data collection

Data collection was based on voluntary registration of BC treatment institutions with the benchmarking system. For each patient, the data for the 173 parameters from the DKG/DGS Requirements of Breast Centres (FAB) and a number of additional parameters were collected by staff members of the participating institutions from 1 January, 2003 to 31 December, 2007. The data were initially recorded using primarily the database-driven oncological documentation system ODSeasy (asthenis^® ^GmbH, Aschheim, Germany). Data collection later used a generic XML-based data set (see below), as the XML technology enables data to be collected from different types of documentation systems. Anonymised (coded) and encrypted data sets were stored on CD-ROM and submitted to the WBC/DOC for independent external analysis twice a year.

### Quality indicators

The 173 individual parameters from the DKG/DGS requirements (FAB) were grouped to yield clinically based QIs designed to determine the degree of achievement of predefined quality targets. The QIs were reviewed once a year, at which time existing QIs were modified, or discontinued if they lacked discriminatory power, and new indicators or subindicators were added, as deemed appropriate by the scientific advisory board.

Methodologically, the QIs used in our study were rate-based indicators with a primary focus on the generally recognised necessity of process quality in terms of compliance with treatment guidelines and methods of decision-making. Process QIs (PQIs) served as short-term **surrogate parameters** for outcome quality, until the accrual of sufficient data over period of 5 to 10 years would permit calculation of long-term outcome QIs, the ultimate objective of the project as a whole.

### Plausibility checks and monitoring

All data from the participating institutions were centrally collected and analysed at the WBC/DOC, and the results of the analysis were reported back twice a year. Data plausibility was ensured by means of twice yearly monitoring visits and data reviews at the annual participants' meetings.

The primary purpose of the monitoring visits was to check the electronic documentation extracted from patients' medical records for correctness and completeness in order to ensure that the database would be valid and suitable for analysis according to the benchmarking procedure. In addition, the monitoring visits were also intended as opportunities to discuss, and advise on, issues related to the documentation process [7,8].

### Generic XML-based data set

To eliminate problems associated with different tumour documentation systems being used as the number of participating institutions rose over the study period, it soon became necessary to develop a generic XML-based data set that would readily integrate with various software systems. In their role as the leading scientific organisations for BC in Germany, the DKG (German Cancer Society) and DGS (German Society of Senology) determined the contents and calculation algorithms of the XML data set on the basis of the level-3 guidelines on early detection of breast cancer in Germany, the interdisciplinary S3 guidelines for the diagnosis and treatment of breast cancer in women and the DKG/DGS Requirements of Breast Centres (FAB) [3,9,5].

To harmonise and standardise the clinical parameters and validate software providers in the context of breast centre certification to the DKG/DGS requirements, the WBC/DOC (West German Breast Centre/German Oncology Centre) was commissioned as an independent external service provider to program a suitable XML-based data set comprising the algorithms for calculation of the QIs.

The XML data set with the algorithms were integrated with the participating institutions' various tumour documentation systems by the respective software providers to ensure the availability of uniform documentation options and QI calculations for the DKG/DGS questionnaire on all computer systems and the implementation of nationwide standards for data generation and data assessment. The programming of the XML data set was overseen by the DKG/DGS and the AGO (DGGG Working Group on Gynaecological Oncology).

### Data analysis and reporting

Data analysis was performed by the WBC/DOC with the aid of standard software, including Access^®^, Excel^® ^and Word^® ^from Microsoft Office 2002/2003 and Microsoft SQL Server 2005 (Microsoft Corporation, Redmond, WA, USA). The query logic was written in SQL and therefore was also compatible with other software products.

For each participating institution and QI, the mean annual percentage of all patients was calculated who were diagnosed or treated in accordance with the relevant QI requirements. For each QI, the participating institutions were then ranked by calculated mean annual percentage, and an overall mean annual percentage was calculated across all participating institutions. In addition, 95% confidence intervals were calculated, which are indicative of the size of the underlying population.

Changes over time in the quality indicators were analysed for the 2003–2007 period using descriptive numerical and graphical methods (tables and histograms). No statistical tests were employed.

The WBC/DOC (West German Breast Centre/German Oncology Centre) provided the participating institutions with six-monthly reports on their individual performance in comparison with the other participants. Semi-annual and annual overall reports in which all hospitals were coded for anonymity were compiled, distributed within the network and also made publicly available online at .

Further details of the methodology are provided in the WBC's annual reports for 2003 to 2007, which are available online at  (in German) [7,8,10-12].

## Results

### Participating institutions, monitoring visits and case numbers

**Figure 1:**
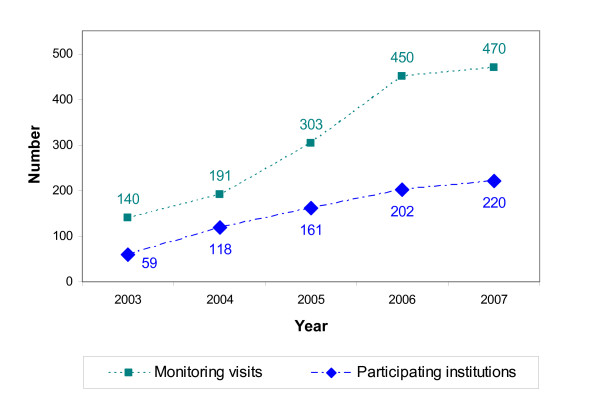
Number of breast centres participating in the benchmarking of breast cancer care and number of monitoring visits during 2003–2007.

As of 2003, a growing number of hospitals and specialist breast centres with a focus on BC care in Germany and, as of 2006, Switzerland entered into formal collaboration with the WBC/DOC (West German Breast Centre/German Oncology Centre) to participate in the voluntary, external and independent scientific benchmarking system operated by the WBC/DOC. This is illustrated by Figure 1, which also shows the accompanying rise in the number of visits per year (median 2.14, range 1.62–2.37) by specially trained monitors to the participating institutions during the 2003–2007 period.

**Figure 2:**
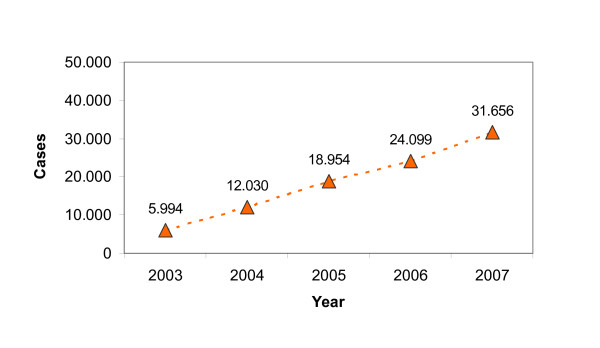
Histologically confirmed primary breast cancers reported by the participating institutions during 2003–2007.

The number of primary BC cases per year as confirmed by postoperative histology also showed a steady increase during the 2003–2007 period, as shown in Figure 2. This marked, 5.28-fold increase in histologically confirmed primary BC cases accompanied a 3,73-fold increase in the number of participating institutions achieved during the five-year study period, reflecting a steady rise in the number of postoperatively confirmed primary BC cases per participating institution from 101.59 in 2003 to 143.8 in 2007.

### XML-based data set

A generic XML-based data set was successfully developed that readily integrated with various types of tumour documentation software and enabled uniform data collection and data analysis. The contents of the XML data set and the embedded calculation algorithms were determined by the DKG (German Cancer Society) and DGS (German Society of Senology) on the basis of the DKG/DGS Requirements of Breast Centres (FAB) [3] and thus were essentially based on the two relevant level-3 guidelines [9,5].

In addition to encompassing the 173 DKG/DGS Requirements of Breast Centres relevant to DKG/DGS certification, the custom-designed, XML-based data set structure also accommodated data relevant to EUSOMA (European Society of Breast Cancer Specialists) certification of breast centres [13] as well as additional items required by German law.

The XML data set and the embedded algorithms were used successfully to ensure uniform documentation and calculation of QIs and the implementation of nationwide standards for data generation and data assessment.

### Quality indicators (QIs)

Starting in 2003 from a set of 9 QIs (Nos. 1, 2, ex-3, 5, 6, 7.1 b, 9, 10 and 11 b in Table 1) derived from the clinical parameters laid down in the DKG/DGS Requirements of Breast Centres (FAB), QIs were reviewed once a year and modified, expanded or discontinued on the advice of the scientific advisory board. Table 1 shows the set of 12 QIs and 9 subindicators used in 2007 to benchmark breast cancer care, listing each QI with its year of introduction, rationale and quality target and the relevant DKG/DGS requirements for years 1 and 3 of the DKG/DGS certification procedure. The QIs identified for the new, nationwide benchmarking system cover a number of crucial aspects of the treatment chain process, ranging from preoperative (QI No. 1) and operative (Nos. 2–4) aspects to breast conserving therapy (BCT) (No. 11), hormone receptor assessment (No. 5) and endocrine therapy (No. 6), neoadjuvant and adjuvant chemotherapy as standard regimens (Nos. 7.1 and 7.2) and study regimens (No. 8) and radiotherapy after BCT or mastectomy (No. 9 and 10). In addition to these process-based surrogate QIs, the rate of relapse-free survival (RFS) has now been identified as the first true indicator of outcome quality to be implemented. However, RFS data are not expected to be available until 2009 at the earliest.

**Table 1:** Quality indicators (QIs) used in the new, nationwide system for benchmarking breast cancer care in 2007

**QI No.**	**Quality indicator (QI)**	**Introduced**	**Based on**	**Quality target**	**DKG/DGS (FAB) requirement**
**1**	**Preoperative histological confirmation of diagnosis**	2003	L3-GL/ED-BC (2003)	Frequent preoperative histological confirmation of diagnosis in invasive breast cancer	> 90% (palpable tumours), > 70% (nonpalpable tumours)

**2**	**Appropriate axillary dissection**	2003	L3-GL/DT-BC (2004)	Appropriate axillary dissection in all patients with invasive breast cancer (axillary clearance)	> 85% at initial certification; > 95% after 3 years

***[ex-3]*^a^**	***Complete tumour staging data***	*2003*	*L3-GL/DT-BC (2004)*	*Complete information on tumour stage (T-N-M-R-G) for all patients*	*> 95% for pT and pN in invasive BC*

**3**	**Data on safety distance between tumour and resection margin**	2007	L3-GL/DT-BC (2004)	Data on safety distance for all patients	Pathologist's report must state the resection margin and minimum safety distance in 100% of cases (exceptions require justification)

***[ex-4]*^a^**	***HER-2/neu assessment***	*2005*	*Generally accepted criterion*	*Frequent assessment of HER-2/neu status*	*> 95% in invasive BC*

**4**	**Specimen radiography**	2007	Generally accepted criterion	Specimen radiography after preoperative wire localisation	Postoperative specimen radiography of microcalcifications following preoperative wire localisation in > 95% of cases

**5**	**Hormone receptor assessment**	2003	L3-GL/DT-BC (2004)	Assessment of hormone receptor status in all patients	100% (except in justified cases)

**6**	**Appropriate endocrine therapy in hormone receptor-positive patients**	2003	L3-GL/DT-BC (2004)	Endocrine therapy in all hormone receptor-positive patients	> 70% at initial certification; > 95% after 3 years

**7.1**	**Appropriate adjuvant and neoadjuvant chemotherapy**	2003	L3-GL/DT-BC (2004)	Frequent appropriate adjuvant or neoadjuvant chemotherapy in breast cancer patients with negative hormone receptor status, or with ≥ 4 affected lymph nodes irrespective of receptor status	See 7.1a and 7.1b
7.1a	during the current analysis period; age ≤ 70 years	2005	L3-GL/DT-BC (2004)	See QI 7.1	> 70% at initial certification; > 80% after 3 years in patients ≤ 70 years
7.1b	during the current analysis period; no age limit	2003	L3-GL/DT-BC (2004)	See QI 7.1	n. d.

**7.2**	**Use of appropriate standard regimens in chemotherapy**	2005	n. d.	Frequent use of appropriate standard regimens in chemotherapy	n. d.
7.2a	during the current analysis period; age ≤ 70 years	2006	n. d.	See QI 7.2	n. d.
7.2b	during the current analysis period; no age limit	2005	n. d.	See QI 7.2	n. d.

**8**	**Percentage of patients in clinical trials**	2005	L3-GL/DT-BC (2004)	Frequent inclusion of patients in clinical trials	≥ 10% and ≥ 20% primary breast cancers at initial certification and after 3 years, respectively

**9**	**Appropriate radiotherapy after breast-conserving therapy**	2003	L3-GL/DT-BC (2004)	Appropriate radiotherapy for all patients receiving breast-conserving therapy	Complete record of the number of radiation treatments; exceptions require justification

**10**	**Appropriate radiotherapy after mastectomy**	2003	L3-GL/DT-BC (2004)	Appropriate radiotherapy for all mastectomy patients	Complete record of the number of radiation treatments; exceptions require justification

**11**	**Indication for breast-conserving therapy**	2003	L3-GL/DT-BC (2004)	Appropriate indication for breast-conserving therapy in all patients	Breast-conserving surgery for pT1 tumours; > 50% at initial certification, > 70% after 3 years
11a	at any tumour stage	2003	L3-GL/DT-BC (2004)	See QI 11	n. d.
11b	at T1	2005	L3-GL/DT-BC (2004)	See QI 11	Breast-conserving surgery for pT1 tumours; > 50% at initial certification, > 70% after 3 years
11c	at T2	2006	L3-GL/DT-BC (2004)	See QI 11	n. d.
11d	at T3	2006	L3-GL/DT-BC (2004)	See QI 11	n. d.
11e	at T4	2006	L3-GL/DT-BC (2004)	See QI 11	n. d.

DKG = Deutsche Krebsgesellschaft (German Cancer Society); DGS = Deutsche Gesellschaft für Senologie (German Society of Senology)

L3-GL/ED-BC (2003) = Level-3 Guidelines for the early detection of breast cancer in Germany (2003)

L3-GL/DT-BC (2004) = Interdisciplinary S3 guidelines for the diagnosis and treatment of breast cancer in women (2004)

^a ^*Square brackets and italics indicate QIs which were discontinued at the end of 2006*; n. d. = no details.

Numbering and names of current QIs and their year of introduction, rationale, quality target and DKG/DGS requirements for years 1 and 3 of DKG/DGS certification.

In the context of the present analysis, the DKG/DGS (German Cancer Society/German Society of Senology) requirements served as target values. No DKG/DGS requirement applied strictly to QIs 7.1b, 11.a and 11.c-e, as these were not restricted to age ≤ 70 years. QIs Nos. 7.1b, 7.2a, 7.2b pertaining to the use of appropriate standard regimens in chemotherapy were not directly derived from the relevant level-3 guidelines but were introduced on the advice of the scientific advisory board. The two QIs which were abandoned at the end of 2006 and are designated "ex-3" and "ex-4" in Table 1 have been included to illustrate how the benchmarking procedure provides for the scientific advisory board to discontinue QIs no longer considered to offer sufficient discriminatory power.

In an attempt to allow hospitals more time to complete entry of e.g. adjuvant and neoadjuvant chemotherapy data into their computer systems, previous-year QIs were introduced in 2006 (not shown) [8]. The rationale of such QIs was that they would contribute to a more complete and accurate database and ultimately enhance the validity of the analysis. However, these subindicators proved unsatisfactory and were abandoned again in 2007. Instead, a general 90-day lag was introduced to allow for completion of data entry.

### QI development during the 2003–2007 period

The results for all QIs evaluated in 2007 are summarised in Table 2, which also shows how the indicators developed during the 2003–2007 period and exemplifies the adaptability of the benchmarking system in terms of enabling addition or discontinuation of QIs. In particular, Table 2 illustrates the addition of new QIs from 2005 to 2007 and the replacement of Nos. "ex-3" and "ex-4" by the new Nos. 3 and 4 in 2007.

**Table 2:** Changes over time in the quality indicators used for benchmarking in 2007

								**DKG/DGS requirement**	
								
**QI No.**	**Quality indicator (QI)**	**Year of introduction**	**2003**	**2004**	**2005**	**2006**	**2007**	**1st year**	**3rd year**
**1**	**Preoperative histological confirmation of diagnosis**	2003	58%	71%	78%	84%	**88%**	90%^a^	90%^a^
**2**	**Appropriate axillary dissection**	2003	85%	85%	80%	83%	**88%**	85%	95%
***[ex-3]*^b^**	***Complete tumour staging data***	*2003*	*85%*	*96%*	*98%*	*95%*		*> 95%*	*> 95%*
**3**	**Data on safety distance between tumour and resection margin**	2007					**91%**	100%	100%
***[ex-4]*^b^**	***HER-2/neu assessment***	*2005*			*94%*	*98%*		*> 95%*	*> 95%*
**4**	**Specimen radiography**	2007					**83%**	> 95%	> 95%
**5**	**Hormone receptor assessment**	2003	92%	96%	96%	97%	**98%**	100%	100%
**6**	**Appropriate endocrine therapy in hormone receptor-positive patients**	2003	27%	82%	92%	94%	**93%**	70%	95%
**7.1**	**Appropriate adjuvant and neoadjuvant chemotherapy**	2003							
7.1a	during the current analysis period; age ≤ 70 years	2005			65%	75%	**81%**	70%	80%
7.1b	during the current analysis period; no age limit	2003	32%	45%	55%	63%	**80%**	--	--
**7.2**	**Use of appropriate standard regimens in chemotherapy**	2005							
7.2a	during the current analysis period; age ≤ 70 years	2006				65%	**72%**	--	--
7.2b	during the current analysis period; no age limit	2005			57%	60%	**69%**	--	--
**8**	**Percentage of patients in clinical trials**	2005			8%	7%	**7%**	10%	20%
**9**	**Appropriate radiotherapy after breast-conserving therapy**	2003	20%	46%	60%	70%	**79%**	100%^c^	100%^c^
**10**	**Appropriate radiotherapy after mastectomy**	2003	8%	26%	35%	47%	**65%**	100%^c^	100%^c^
**11**	**Indication for breast-conserving therapy**	2003							
11a	at any tumour stage	2003	64%	66%	64%	68%	**70%**	--	--
11b	at T1	2005			79%	83%	**85%**	50%	70%
11c	at T2	2006				60%	**63%**	--	--
11d	at T3	2006				15%	**13%**	--	--
11e	at T4	2006				15%	**12%**	--	--

^a ^requirement for palpable tumours, 70% for nonpalpable tumours

^b ^*Square brackets and italics indicate QIs which were discontinued at the end of 2006*

^c ^number of radiation treatments to be recorded; exceptions require justification

-- no specified requirement.

Numbering and names, year of introduction, QI values for the 2003–2007 period (mean percentages across all participating institutions) and the required percentages for years 1 and 3 of DKG/DGS certification.

Among the eight indicators tracked throughout the study period from 2003 to 2007, Nos. 1, 6, 7.1b, 9 and 10 showed marked increases, while the others exhibited little change (Nos.5 and 11a) or practically no (No. 2) change. Among the seven indicators tracked for at least two years during the study period, moderate increases were seen for Nos. ex-3, 7.1a and 7.2b, while little change was noted in Nos. ex-4, 7.2a and 11.b, and No. 8 remained practically unchanged.

**Figure 3:**
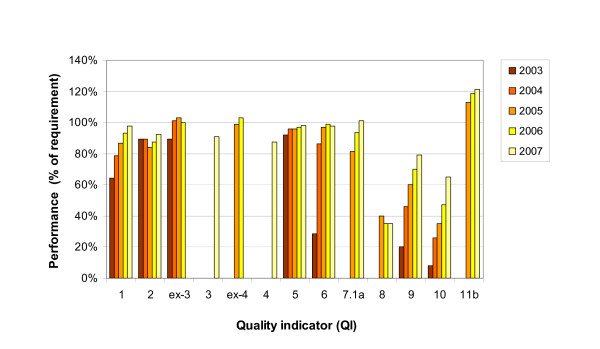
**Performance of quality indicators (QIs) compared to the respective DKG/DGS Requirements of Breast Centres (FAB) during the 2003–2007 period**. QIs Nos.: 1 = Preoperative histological confirmation of diagnosis; 2 = Appropriate axillary dissection; [ex-3] = Complete tumour staging data; 3 = Data on safety distance between tumour and resection margin; [ex-4] = HER-2/neu assessment; 4 = Specimen radiography; 5 = Hormone receptor assessment; 6 = Appropriate endocrine therapy in hormone receptor-positive patients; 7.1a = Appropriate adjuvant and neoadjuvant chemotherapy during the analysis period, age ≤ 70 years; 8 = Percentage of patients in clinical trials; 9 = Appropriate radiotherapy after breast-conserving therapy; 10 = Appropriate radiotherapy after mastectomy; 11b = Indication for breast-conserving therapy at T1. QI No. 1 *(Preoperative histological confirmation of diagnosis) *was compared against the stricter DKG/DGS requirement of 90% (for palpable tumours as opposed to 70% for nonpalpable tumours) as the benchmark. The benchmarking system does not currently distinguish between palpable and nonpalpable tumours. QIs labelled "ex-3" *(Complete tumour staging data) *and "ex-4" *(HER 2/neu assessment) *were discontinued at the end of 2006 and replaced by QIs "3" *(Data on safety distance between tumour and resection margin) *and "4" *(Specimen radiography) *in 2007. Relative performance was not defined for QIs 7.1b, 7.2a, 7.2b, 11a and 11.c-e in the absence of relevant DKG/DGS requirements.

For those QIs representing parameters for which the DKG/DGS Requirements of Breast Centres (FAB) specified target values, performance levels were calculated relative to the third-year DKG/DGS certification requirements. These were stricter than the first-year requirements in the cases of QIs Nos. 2, 6, 7.1b, 8 and 11b. The resulting QI performance data are shown in Figure 3. They clearly illustrate for most QIs how individual indicators developed over the 2003–2007 period. For instance, the performance levels of QIs Nos. 1 (preoperative histology) and 6 (endocrine therapy in receptor-positive patients), were low (58%) or very low (27%) in 2003, but improved to high levels around 90% by 2007. Nos. 9 (radiotherapy after BCT) and 10 (radiotherapy after mastectomy) increased from very low levels (20% and 8%) to high (79%) or intermediate (65%) levels, respectively. QIs Nos. 2 (axillary dissection), ex-3 (complete tumour staging data), 5 (hormone receptor assessment) and 7.1a (adjuvant and neoadjuvant chemotherapy at age ≤ 70 years) had high initial levels and either remained essentially unchanged at about the first-year DKG/DGS level (No. 2) or improved further to achieve levels close to (Nos. ex-3 (by 2006) and 5) or in excess of 100% (No. 7.1a). The relative performance of QI No. 11b (BCT at T1) was consistently above 100% from 2005, the year of its introduction, until 2007, as it always exceeded the DKG/DGS requirement that BCT be recommended in at least 70% of patients with T1 tumours.

## Discussion

Breast cancer continues to be the most common cancer in women in Germany and throughout the world [14,15]. With more than 55,100 women estimated to be diagnosed with breast cancer annually, Germany ranked ninth in a 2002 comparison of the incidence rates of BC in 24 European countries [15].

In recent years, health policies in Germany have underscored the increasing importance attributed to breast cancer [16,17]. Efforts have been directed towards developing and implementing structured, intersectoral quality management programmes aimed at optimising breast cancer care to reduce inappropriate care and the over- and underprovision of care [18].

Until the initiation of the present study in 2003, however, no suitable software, necessary infrastructure or benchmarking system with specific collaboration agreements existed either at the national or international level.

Moreover, despite the clear need for benchmarking systems to assess cancer care, literature searches conducted up to 2007 yielded no evidence indicating that mandatory or voluntary supraregional inter-institutional benchmarking systems existed in other countries for collecting and analysing data on the quality of care provided to patients with breast cancer, or other cancers. This shows the novelty of the approach that has been pursued in Germany since 2003 with the aim of creating a national benchmarking network of breast centres. To the best of our knowledge this is the first publication in the international literature to report on the implementation of, and present initial data from, a comprehensive nationwide QA system for benchmarking the quality of cancer care along the entire process from diagnosis to follow-up.

The objectives of the present proof-of-concept study were fourfold: 

1. to demonstrate the feasibility of QA in breast cancer care by establishing a voluntary benchmarking network of breast centres in collaboration with an external, independent service provider commissioned to collect and analyse the QA data;

2. to identify and define QIs based on clinical parameters in accordance with the criteria laid down by the relevant scientific medical societies;

3. to develop an XML data set designed to enable uniform collection and analysis of the participants' QA data; and finally,

4. to determine whether and to what extent such a benchmarking system would be able to reveal QI-specific differences between the (anonymised) participating institutions and to quantify the QI changes across the network as a whole over the first five-year period from 2003 to 2007.

The marked year-to-year increases in** breast centre participation**, the resulting 3.7-fold overall increase in participating centres from 59 in 2003 to 220 by the end of 2007 and the associated rise in the number of annual external **monitoring visits** shown in Figure 1 clearly demonstrate that voluntary collection and analysis of QA data for benchmarking the quality of breast cancer care based on self-declaration rather than legislation was both feasible and well accepted by the treatment centres. With a median 2.14 monitoring visits per year, the monitoring data were also consistent with the planned number of 2 visits per year.

The substantial, 5.28-fold increase in the number of **histologically confirmed primary breast cancers** reported to the WBC/DOC (West German Breast Centre/German Oncology Centre) from 5,994 in 2003 to 31,656 in 2007 also clearly indicates the high level of acceptance among the participating centres for the voluntary collection of QA data for benchmarking and the success of the benchmarking system as a whole. Whereas our benchmarking system accounted for some 15% of breast cancer cases in Germany in 2003, the more than 31,000 new cases of histologically confirmed breast cancer reported in 2007 represent about 63% of the 50,100 new breast cancers estimated to occur in Germany every year [10,8].

As regards annual caseload, it is worth noting that even in the first year of the study the mean number of histologically confirmed primary breast cancers per participating centre exceeded 100, the volume of new cases found by Hébert-Croteau et al. to be associated with improved survival [19]. In fact, mean annual caseload per centre rose consistently by 41.6% in the present study from 101.6 in 2003 to 143.9 by the end of 2007. This finding could suggest that by the end of 2007, mean caseload was approaching the high annual volume of 150 surgical cases per hospital shown by Roohan et al. to be associated with a significant positive effect on 5-year survival from breast cancer [20]. A plausible interpretation of the observed increases in mean caseload per centre is that they reflect a trend towards centralisation in breast cancer care as a result of the increasing referral of patients to specialist breast centres over the study period, thus indirectly indicating improvement in the quality of BC care.

Lastly, the cumulative **number of data sets** (postoperatively confirmed primary breast cancers) collected by 31 December, 2007 exceeded 92,000.

Taken together, these results provide clear evidence of the willingness among breast centres in Germany, and more recently Switzerland, to engage in self-declaration of QA data relating to breast cancer care. They reflect the project's remarkable success in implementing and maintaining a network for benchmarking breast cancer care and therefore can be considered to provide proof of concept for the feasibility of such a system.

Proof of concept was also established with regard to defining a set of, initially, nine rate-based **quality indicators** (referred to here as Nos. 1, 2, ex-3, 5, 6, 7.1b, 9, 10 and 11a) based on clinical parameters primarily derived from the relevant Level-3 guidelines [9,5] and the DKG/DGS (German Cancer Society/German Society of Senology) Requirements of Breast Centres (FAB) [3]. These initial QIs were process indicators selected to serve as surrogate indicators of outcome quality. As the data accrue over the coming years, true indicators of outcome quality will be introduced, the first of which, disease-free survival (DFS), is in currently preparation.

The successful development of a generic **XML-based data set**, i.e. a software-independent data set structure that readily integrates with any existing tumour documentation system, enabled standardised, uniform collection and analysis of QA data on BC care. This has greatly facilitated, and continues to facilitate, the participation of a still growing number of breast centres in this voluntary benchmarking system designed to evaluate and improve the quality of BC care.

The generic data set structure ensured that the QA data were collected consistently across all participating hospitals and all calculations were performed in exactly the same way using parameter definitions and algorithms laid down by the data advisory board on the basis of the DKG/DGS Requirements of Breast Centres (FAB). Accordingly, the generic XML-based data set ensured the comparability of results across all participating institutions, thus providing proof of concept for the feasibility of uniform data collection and data analysis in the context of the present benchmarking project.

The present study also provided proof of concept for the **feasibility of a benchmarking system** based on the QIs described above in that it proved capable of demonstrating both QI-specific differences between the anonymised individual participating institutions in the form of rankings (data not shown) and **changes in quality indicators** across the network as a whole, thus indicating **improvements in the quality of breast cancer care over the first five-year period from 2003 to 2007**.

The QIs reported here were initially selected with a view, inter alia, to gauging the quality of care being provided to patients with primary breast cancer in Germany at the time in terms of minimum standards and the extent to which they were being met by each individual participating centre and across all centres. As evidenced by Table 2 and Figure 3, the project was successful in identifying an initial set of 9 rate-based QIs, which were reviewed annually and modified, expanded or discontinued. The first results for 2003 showed average BC care as provided by the initial group of 59 participating centres to be reasonably satisfactory, e.g. with regard to axillary dissection (QI No. 2), complete tumour staging data (QI No. ex-3) and hormone receptor assessment (QI No. 5), but in need of improvement in other respects, e.g. endocrine therapy in hormone receptor-positive patients, radiotherapy after breast-conserving therapy and radiotherapy after mastectomy (QIs Nos. 6, 9 and 10, respectively).

The project was also successful in demonstrating that up-to-date QI results could be calculated and reported to the participating institutions twice a year, thus enabling them to react in a timely manner and introduce improvements in the care of their BC patients. In this way, marked progress was achieved with regard to preoperative histological confirmation of diagnosis, endocrine therapy in hormone receptor-positive patients, radiotherapy after breast-conserving therapy and radiotherapy after mastectomy (QIs Nos. 1, 6, 9 and 10, respectively). Overall, moderate to marked improvements were achieved for the vast majority of QIs during the 2003–2007 period. The most notable exception was the percentage of patients in clinical trials (QI No. 8), which failed to achieve the DKG/DGS benchmark value (i.e. the 3^rd^-year FAB requirement) of 20% throughout the study period.

Initial high levels of QI performance (QIs Nos. 2, ex-3 and 5) compared to the relevant DKG/DGS benchmarks, and increases in the performance of most QIs over the first 5-year period strongly suggest that the quality of BC care in Germany has improved considerably since 2003. However, it is clear from Figure 3 that there is still scope for improvement, particularly with regard to QIs Nos. 2, 3, and 4 and even more so, Nos. 8, 9 and 10.

During the study period, the initial set of nine QIs was successively expanded as of 2005 to include new indicators (e.g. No. 8 (2005), 3 and 4 (2007)) and subindicators (e.g. 7.1a (2005), 7.2b (2005) and 7.2a (2006)). Conversely, two QIs (ex-3 (complete tumour staging data) and ex-4 (HER 2/neu assessment)) were abandoned at the end of 2006, because they were no longer considered to possess sufficient discriminatory power to indicate differences in quality. In addition, the criteria for including patients in, or excluding them from, the QI calculations were stated more precisely in several cases. These and other alterations and revisions demonstrate the flexibility and adaptability of the benchmarking system we describe.

A major advantage of the newly implemented benchmarking system is that it permits the collection of **longitudinal data** over any desired period of time. This will enable the introduction of true outcome QIs reflecting the quality of BC care beyond primary hospital care, including such aspects as adjuvant therapies, complications after discharge, disease-free survival, recurrence rates and mortality rates in the near future.

An electronic network is currently being set up in Germany with the aim to establish a comprehensive, nationwide quality management system comprising all breast centres, specialist practices and practising oncologists. This intersectoral network will utilise the generic XML-based data set discussed above to enable external, documentation system-independent collection of all diagnostic, treatment and follow-up data for all breast cancer patients, especially the follow-up data from the specialists in private practice.

The participation of two Swiss hospitals in the reported benchmarking programme strongly suggests that it can also be implemented in countries other than Germany. There may, however, be limitations to transferability due to e.g. potential differences in professional culture, clinical practice or health care system, as recently demonstrated by Marshall et al. [21] in an analysis of 174 QIs covering 18 non-cancer conditions with the highest consultation rates in general practice in the United States compared with the United Kingdom.

Based on the positive experience with benchmarking the quality of BC care reported here, analogous uniform XML-based data sets have recently been developed in Germany for other cancers. In particular, the new benchmarking system has been adapted for measuring the quality of care provided to patients with colon or prostate cancer. This has resulted in the establishment of other site-specific multidisciplinary cancer centres (for colon and prostate cancer, to begin with) under the auspices of the German Cancer Society in collaboration with the German Oncology Centre (DOC).

## Conclusion

The results from the first five years of the present study provide proof of concept for the feasibility of a novel, voluntary, nationwide system for benchmarking the quality of BC care based on collaboration between a network of specialist breast centres and an independent external provider of scientific data collection and data analysis services. The study also provides proof of concept regarding the uniform collection and analysis of the relevant QA data by means of a new, purpose-designed, generic XML-based data set that integrates with any existing tumour documentation software; the definition of clinically based indicators of the quality of BC care; and the practicability of using the quality indicators to measure both within-network differences in quality (ranking), performance compared to defined target values (benchmarking) and changes in the quality of care over time.

The collected data are valid due to the checks performed during the twice-yearly monitoring visits. The QIs identified and reviewed in the context of the annual participants' meetings are sufficiently discriminatory to indicate both differences in quality among the participating institutions and changes in quality which occurred over the first five-year period, 2003–2007. The current QIs can be considered useful surrogate parameters for long-term outcome quality, which are expected to become available as the data accrue over the second five-year period.

After five years, more than 60% of the estimated annual total of 50,000 new cases of breast cancer in Germany can be said to be diagnosed, treated and followed up at one of the now 220 specialist hospitals and breast centres participating in the nationwide system for benchmarking the quality of breast cancer care.

Overall, the new, nationwide benchmarking system has proved a clinically oriented, practical, flexible, adaptable and extensible tool for measuring and improving the quality of breast cancer care during the 2003–2007 study period.

Finally, with two breast centres in Switzerland joining the new benchmarking system in 2006, it has been shown that the collaborative benchmarking network is not per se limited to Germany but can be extended to the supranational level.

## Abbreviations

AGO: Arbeitsgemeinschaft Gynäkologische Onkologie (DGGG Working Group on Gynaecological Oncology); BCT: breast-conserving therapy; BC: breast cancer; DGGG: Deutsche Gesellschaft für Gynäkologie und Geburtshilfe (German Society of Obstetrics and Gynaecology); DFS: disease-free survival; DGS: Deutsche Gesellschaft für Senologie (German Society of Senology); DKG: Deutsche Krebsgesellschaft (German Cancer Society); DOC: Deutsches Onkologie Centrum Holding GmbH (German Oncology Centre Ltd.); EUSOMA: European Society of Breast Cancer Specialists (formerly: of Mastology); FAB: Fachliche Anforderungen an Brustzentren (Requirements of Breast Centres); HER-2/neu: human epidermal growth factor receptor 2; PQI: process quality indicator; QA: quality assurance; QI: quality indicator; SQL: structured query language; WBC: Westdeutsches Brust-Centrum (West German Breast Centre); XML: extensible markup language.

## Competing interests

The authors declare that they have no competing interests.

## Authors' contributions

SYB compiled and analysed the data presented and drafted and finalised the manuscript. CS, ChS and MR reviewed the draft manuscript. MB participated in the development of the benchmarking system described and reviewed the draft manuscript. DW participated in the development of the benchmarking system described, conceived of the present study and its design, outlined the contents and reviewed the draft. All authors read and approved the final manuscript.

## Pre-publication history

The pre-publication history for this paper can be accessed here:


